# Efficacy and safety of ondansetron in preventing postanesthesia shivering: a meta-analysis of randomized controlled trials

**DOI:** 10.1186/1471-2253-14-12

**Published:** 2014-03-03

**Authors:** Hong-Tao Tie, Guang-Zhu Su, Kun He, Shao-Rong Liang, Hao-Wei Yuan, Jun-Huan Mou

**Affiliations:** 1 The First College of Clinical Medicine, the First Affiliated Hospital of Chongqing Medical University, Chongqing, China; 2 Department of Pharmacy, Jinan Central Hospital, Jinan 250013, Shandong, China

**Keywords:** Qndansetron, Postanesthesia shivering, Meta-analysis

## Abstract

**Background:**

Shivering is a very common complication in the postanesthesia period. Increasing studies have reported ondansetron may be effective in prevention of postanesthesia shivering (PAS). However, the results remained controversial; hence we conducted a meta-analysis of randomized controlled trials to evaluate the efficacy and safety of ondansetron on the prevention of postanesthesia shivering.

**Methods:**

PubMed and Embase databases were searched to identify the eligible randomized controlled trials assessing the effect of ondansetron on the prevention of PAS. Results were expressed as risk ratios (RRs) with accompanying 95% confidence intervals (CIs). The meta-analysis was performed with fixed-effect model or random-effect model according to the heterogeneity.

**Results:**

Six trials including 533 subjects were included. Compared with placebo, ondansetron was associated with a significant reduction of PAS (RR 0.43, 95% CI, 0.27-0.70), without an increased risk of bradycardia (RR 0.37, 95% CI, 0.12-1.15). Compared with meperidine, no difference was observed in the incidence of PAS (RR 0.68, 95% CI, 0.39-1.19) and bradycardia (RR 2.0, 95% CI, 0.38-10.64).

**Conclusions:**

Ondansetron has a preventive effect on PAS without a paralleled side effect of bradycardia.

## Background

Shivering, a very common complication of surgery owing to postoperative pain and postanesthesia hypothermia, is distressing for both patients and clinicians [1]. It can be defined as involuntary and oscillatory muscular activities that increase the metabolic rate by two to three folds to maintain the core temperature, with the increment of heat production by only 200% in adults [2]. Both neuraxial (epidural and spinal anesthesia) and general anesthesia are associated with a significant incidence of shivering, and the incidence is 40%-60% in regional anesthetic patients [3] and up to 60% in general anesthetic ones [4]. Shivering can be associated with severe adverse effects by increasing the oxygen consumption and carbon dioxide retention. It can cause arterial hypoxia, increase cardiac output and the risk of myocardial ischemia. Besides, the movement of shivering interferes with the electrocardiogram, blood pressure, and pulse oximetry [5].

Recent years, with increasing awareness of its undesirable aftermath, effective prevention of postanesthesia shivering (PAS) is being imperative. It has been reported that PAS could be prevented by warming skin-surface [6] and warming the administered fluid [7,8], but it is not a perfect way. Many drugs have been shown to be effective on prevention of PAS, such as opioids, α2-agonist, anticholinergic, CNS stimulant, corticosteroid [9], however, few of them were recommended for the prevention of PAS due to various side-effects. For instance, Clonidine, a partial α2 adrenergic agonist, is related to bradycardia, hypotension and sedation [10].

Ondansetron, a 5-TH3 receptor antagonist, is widely used to prevent postoperative and pregnancy nausea and vomiting. 5-TH can affect the body temperature and shivering in rats since the balance of nor-epinephrine and 5-hydroxytryptamine (5-HT) in the preoptic-anterior hypothalamus controls the temperature set point [11,12]. Consistently, several studies [3,13-16] have demonstrated ondansetron can prevent PAS, which made ondansetron a promising drug for postoperative complications including PAS, nausea and vomiting. However, a later-day, large-sample study conducted by Browning et al. [17] presented that ondansetron did not prevent PAS among women undergoing combined spinal and epidural anesthesia for cesarean delivery. To our knowledge, there is no meta-analysis to identify the precise effect of ondansetron on the PAS, therefore, we performed a systematic review and meta-analysis of randomized controlled trials to evaluate and quantify the preventive effect of ondansetron on PAS. Otherwise we also compared ondansetron with meperidine about the effect on PAS and adverse effects.

## Methods

### Literature search and study selection

Potentially relevant studies were identified by searching PubMed and Embase databases through July 2013 with the terms of “ondansetron” and “shivering”. No limitation was applied. In addition, reference lists from identified citations and relevant reviews were manually searched for additional studies. Inclusion criteria for this meta-analysis were as follows: (1) the objects underwent a surgical operation under the neuraxial anesthesia or general anesthesia; (2) the comparison was between ondansetron and placebo or meperidine about the preventive efficacy of PAS; (3) the incidences of PAS were reported in both placebo and ondansetron groups; (4) the study design was randomized clinical trial. No minimum sample sizes were considered for inclusion of studies in the analysis. The disaccords were resolved by discussion.

### Data extraction and quality assessment

Data (patient characteristics, surgical setting, anesthetic type, mean core temperature (°C) at baseline, time of drug administration, definition of PAS, and incidence of PAS) was collected individually by two reviewers. Data was extrapolated from figures as needed. The qualities of the included studies were evaluated by the Jadad scale with the full score of five points. In addition to the primary outcome of PAS; the adverse effects were also extracted from the included studies if possible. Any dispute was resolved by discussion.

### Statistical analysis

The main analysis was focused on the association between ondansetron and PAS, and subgroup analyses stratified by type of anesthesia and dosage of ondansetron were also conducted. Since three included studies [13-15] investigated the prophylactic effect of meperidine on PAS, we also compared ondansetron and meperidine on PAS, and the adverse effects were also explored when available.

Either fixed-effect model or random-effect model was used according to the heterogeneity. Heterogeneity was evaluated by Q statistic (a significant level of *P*<0.1) and *I*^*2*^ statistic (greater than 50% as evidence of a significant level). A two-tailed *P* valueless than 0.05 represents statistical significance in all tests except the specific condition where a *P* value was declared. All statistical analyses were performed with Stata 12.0 software (StataCorp, College Station, TX, USA).

## Results

**Figure 1:**
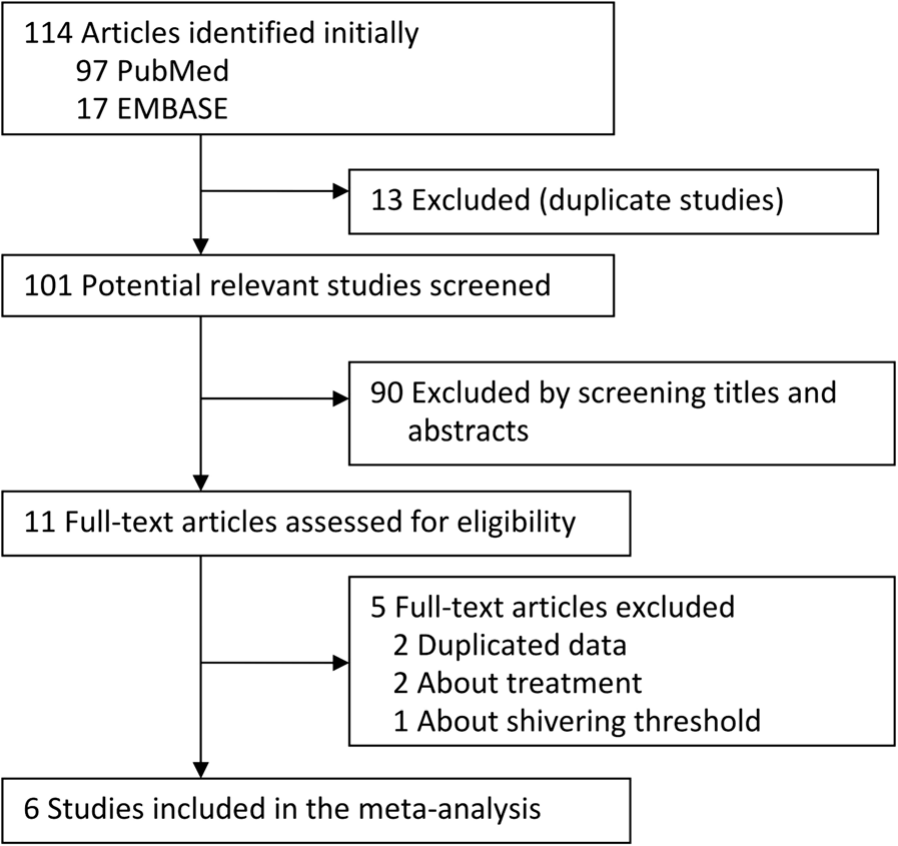
The flow chart of study selection.

114 potential studies (PubMed 17, EMBASE 97) were obtained. The detailed screening flow was shown in Figure 1. After screening the titles and abstracts, 103 studies were excluded because they were duplicates or did not provide available data. Five studies were excluded by full-text screening, of which two [18,19] were duplicates, two [20,21] aimed to investigate the therapeutic effect of ondansetron on PAS, the other one [22] was to determine the effect of ondansetron on threshold of shivering. Detailed characteristics of 6 included studies in this meta-analysis are presented in Table 1.

**Table 1:** The characteristics of the included 6 studies in this meta-analysis

**Study**	**Study design/Jadad score**	**Patient characteristics; surgical setting**	**Type of anesthesia**	**Comparisons (no. patients)**	**Mean temperature (°C) at baseline**	**Time of administration**	**Definition of PAS**
Powell et al. [16]	Randomized, placebo-controlled, double-blinded/ 5	18-60 yr, ASA: I – II; orthopedic, general, or urological surgery	General	Ondansetron IV 4 mg (27)	36.9	3–5 min before the induction of anesthesia	Readily detectable fasciculations or tremors of the face, trunk, or limbs of a minimum of 15-s duration
				Ondansetron IV 8 mg (27)	36.7		
				Saline IV (28)	36.7		
Kelsaka et al. [15]	Randomized, placebo-controlled, double-blinded/ 3	20-60 yr, ASA: I – II; elective orthopedic surgery	Spinal	Ondansetron IV 8 mg (25)	36.9	Immediately before spinal anesthesia	Pectoralis, major muscles for fasciculations more than 10 seconds’ duration.
				Meperidine IV 0.4 mg/kg (25)	36.9		
				Saline IV (25)	36.7		
Shakya et al. [3]	Randomized, placebo-controlled/2	Age > 18 yr, ASA: I – II; elective lower abdominal surgery	Spinal	Ondansetron IV 4 mg (40)	36.7	Just after the intrathecal injection	0 = no shivering. 1 = piloerection or peripheral vasoconstriction but no visible shivering. 2 = muscular activity in only one muscle group. 3 = muscular activity in more than one muscle group but not generalized. 4 = shivering involving the whole body.
				Saline IV (40)	36.8		
Entezari et al. [14]	Rrandomized, placebo-controlled, single-blind/3	Age > 18 yr, ASA: I – II; elective gynecological surgery	General	Ondansetron IV 4 mg (30 )	37.5	2 minutes before inducting anesthesia	Chills for at least 15 seconds
				Meperidine IV 0.4 mg/kg (30)	37.4		
				Saline IV (30)	37.5		
Abdollahi et al. [13]	Randomized, placebo- controlled, double-blind/3	All ages, ASA: I – III; off-pump coronary artery bypass graft	General	Meperedine IV 0.4 mg/Kg (30)	NA	15 minutes before the end of surgery	0 = Patient with no shivering. 1 = whenever one of these symptoms is present: peripheral vasoconstriction, peripheral cyanosis without any other reason and piloerection without muscle contraction. 2 = Visible muscular activity that involved only one muscle group. 3 = Visible muscular activity that involved more than one muscle group. 4 = Intensive muscular activity that involved the whole body.
				Ondansetron IV 8 mg (30)	NA		
				Saline IV (30)	NA		
Browning et al. [17]	Randomized, placebo-controlled, double-blinded/5	Age > 18 yr, ASA: I – II; elective cesarean surgery	Combined spinal epidural anesthesia	Ondansetron IV 8 mg (56)	36.6	Before anesthesia	0 = no shivering. 1 = or more of the following: piloerection, peripheral vasoconstriction, peripheral cyanosis without other cause but without visible muscular activity. 2 = visible muscular activity confined to 1 muscle group. 3 = visible muscular activity in more than 1 muscle group. 4 = gross muscular activity involving the entire body.
				Saline IV (60)	36.8		

Totally 533 subjects were involved in the 6 included trials, of which 235 received ondansetron, 213 received a placebo, and 85 received meperidine. The dosage of ondansetron was either 4 mg or 8 mg in individual study, and meperidine was 0.4 mg/kg in all the three studies [13,15,18]. The trial drugs were all given before anesthesia, but one [17] at the end of the surgery.

**Figure 2:**
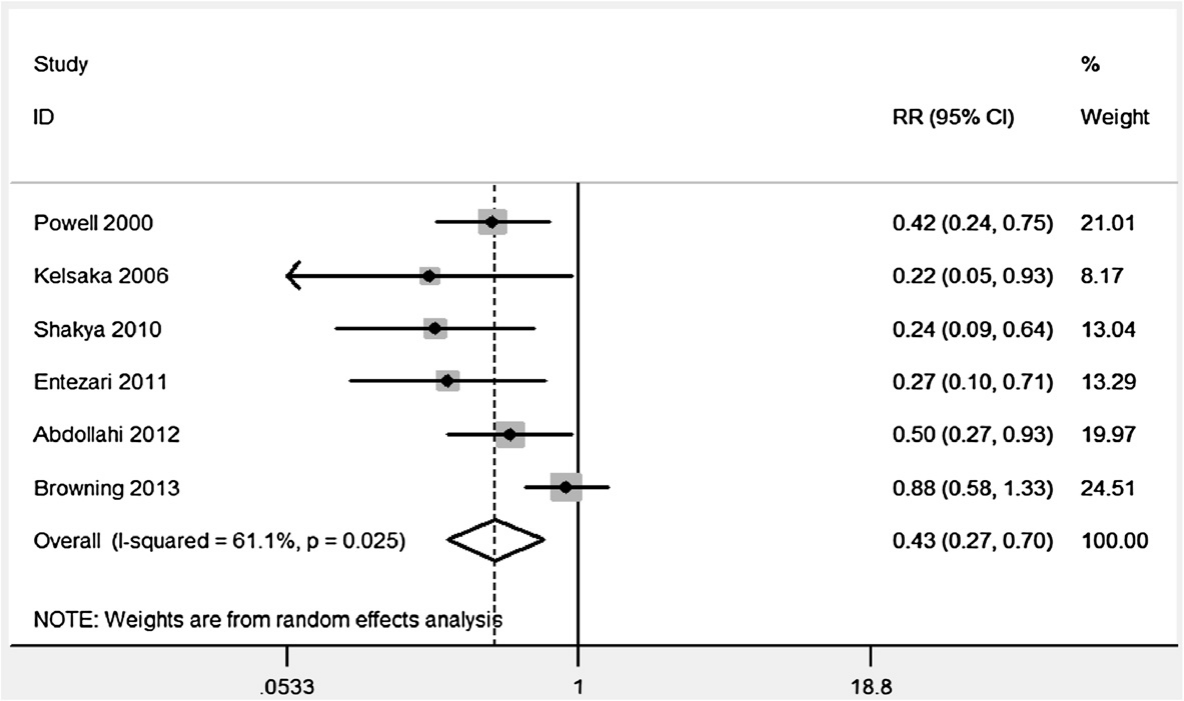
The pooled estimate of the 6 included studies by random effect model.

**Figure 3:**
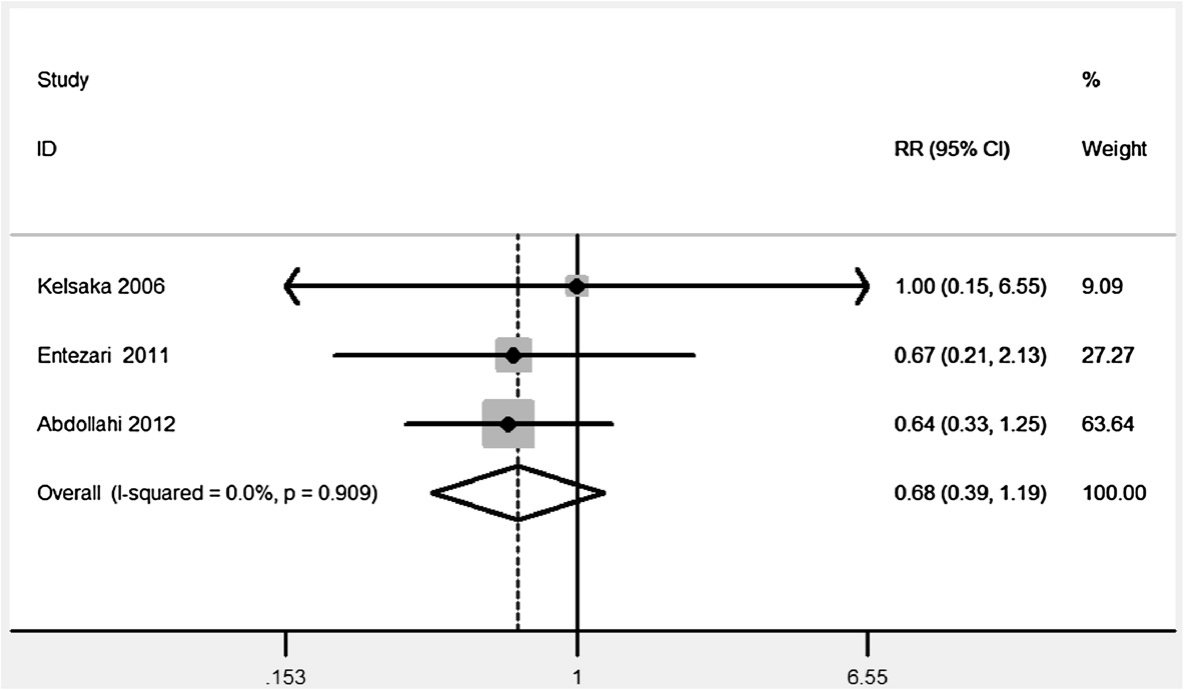
The antishivering effect of ondansetron comparing with meperdine.

**Figure 4:**
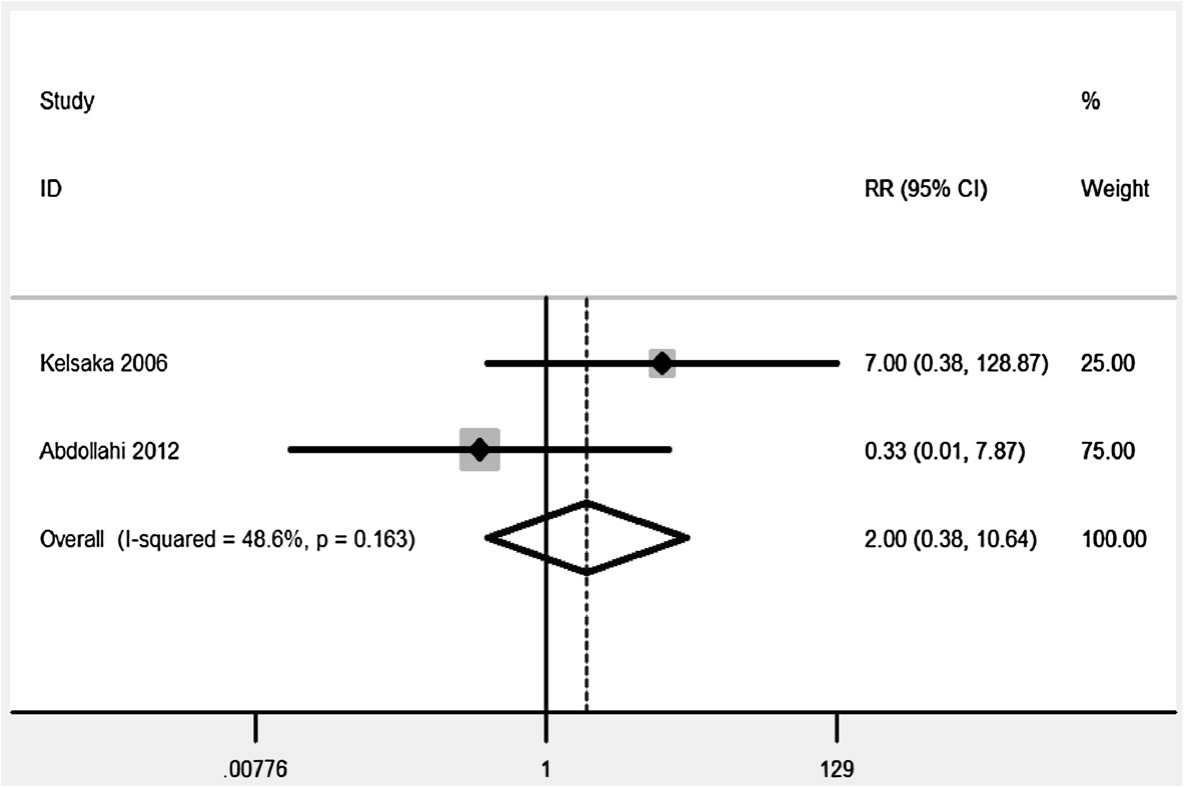
The side effects of bradycardia of ondansetron by comparing with meperdine.

The pooled estimate suggested that ondansetron had a potential effect on the prevention of PAS (RR = 0.43, 95% CI, 0.27-0.70) (Figure 2), with a moderate evidence of heterogeneity (*I*^*2*^ = 61.1%, *P* = 0.025). Results of subgroup analyses were showing in Table 2, and all demonstrated that ondansetron was associated with a significant reduction of PAS risk.Additionally, the adverse effect and comparison with meperidine of ondansetron were analyzed with a limited test power. Compared to placebo, no significant association of ondansetron with bradycardia was found (RR = 2.00, 95%, 0.38-10.64). Significant differences of ondansetron were also not observed in PAS (RR = 0.68, 95% CI, 0.39-1.19) and bradycardia (RR = 2.0, 95% CI, 0.38-10.64) by comparison with meperidine (Figures 3 and 4).

**Table 2:** Results of subgroup analysis by dosage of ondansetron and anesthetic techniques

**Group**	**No. of studies**	**Relative risk**	**I**^**2**^	**P**_**heterogeneity**_	**Effect model**
**Dosage**					
4 mg	3	0.36 (0.22, 0.58)	40.7	0.185	Fixed
8 mg	3	0.47 (0.25, 0.90)	66.0	0.032	Random
Anesthetic technique					
General	3	0.40 (0.27, 0.60)	0	0.559	Fixed
Spinal	2	0.23 (0.10, 0.52)	0	0.949	Fixed

## Discussion

According to our findings, compared with placebo, ondansetron could reduce the prevalence of PAS from 49.3% (placebo) to 23.4% without increased risk of bradycardia. However, No superiority was observed in ondansetron over meperidine on the effect of antishivering and incidence of bradycardia.

Serotonin system plays an important role in the thermoregulation. The mechanisms of ondansetron on PAS may be related to a central mechanism of the inhibition of 5-HT reuptake on the preoptic anterior hypothalamic region [15]. It was proven that 5-HT3 agonist could induce hyperthermia, and its antagonist could lead to hypothermia [23,24]. However, in healthy volunteers, the core-temperature thresholds triggering shivering did not change under comparison with placebo [22]. Several studies [15,16,25] have proven that core-to-peripheral redistribution of body temperature after the general anesthetics is characterized by 1 decrease in the core temperature at the first 20 to 30 minutes, and this alteration kept similar in ondansetron group [15,16]. The evidences above suggested that the effect of ondansetron on the prevention of PAS is irrelevant to the core hypothermia. On the contrary, Kelsaka et al. [15] found that the core temperature was preserved in ondansetron group rather than placebo, but no one shared the same idea with them. So there is little evidence supporting preserving the temperature to be another antishivering mechanism.

Shivering differs from general anesthesia to neuraxial anesthesia. General anesthesia could impair the central thermoregulation, while neuraxial anesthesia impairs both central and peripheral thermoregulation, by enlarging interthreshold range via raising the sweating threshold and decreasing the vasoconstriction and shivering thresholds [26]. The core temperature decrease will be in a plateau after 3-4 h in general anesthesia but no plateau appears in the neuraxial anesthesia, because the vasoconstriction will be evoked when the core temperature triggers the reset vasoconstriction threshold in general anesthesia but not neuraxial anesthesia [2]. Thus, more heat will be lost, and more incidences will occur in neuraxial anesthesia. However, in our analysis, there was no difference in the risks of PAS between general and neuraxial anesthesia. It might be explained by short duration of the operation, and limited sample sizes, different heat preservation measures after operation.

As for dose-dependent effect of ondansetron on shivering, Powell et al. [16] found that ondansetron was associated with a dose-dependent reduction in shivering, while the effect was not observed in pooled effect. As we can see the incidence of shivering was 10% in people weighed about 52 kg with low-dose ondansetron (4 mg) used [15], but Powell et al. [16] found the incidence was 8% in people weighed about 76 kg with high-dose ondansetron(8 mg). So we presume the various weights of the subjects might mask the dose-dependent effect. When compared to other drugs, ondansetron is absent from the hemodynamic effect in the treatment and prevention of PAS [15,26,27], which contributes much to its safety. Thus, the result that no difference exists in bradycardia between ondansetron and placebo is acceptable despite a limited sample. Additionally, two fresh studies demonstrated ondansetron is safe for pregnant women and fetus [28,29]. Apart from the primary outcome of PAS and side effect of bradycardia, other side effects were also mentioned but were not appropriate for quantitative analysis, among which none indicated a significant risk of convulsion, myoclonus, rush, pruritus, headache, pain, hypotension, sedation, nausea or vomiting.

Meperidine has a therapeutic effect on PAS, and its mechanism is likely to be associated with activation of κ receptor [30-32]. In our meta-analysis, we compared the effect of meperidine and ondansetron on the prevention of PAS, no significant superiority was found in ondansetron. While taking the adverse effects into consideration, accumulating studies show that meperidine could increase the incidence of nausea and vomiting [30,31] and induce respiratory depression [16]. Like ondansetron, meperidine at dose of 0.4 mg/kg for treatment of PAS rarely causes cardiovascular effects, which were also poorly confirmed by our combined estimate with small sample.

Many drugs aimed at the treatment and prophylactic of PAS have been found. Physostigmine inhibits PAS through cholinergic system, but it would also cause nausea and vomiting, increased heart rate and blood pressure [33]. Tramadol, which could inhibit 5HT-uptake and increase its release, reduces PAS after general anesthesia [34]. In contrast to ondansetron, it could depress the thresholds of sweating, vasoconstriction and shivering [35]. Doxapram, used as a stimulant in respiratory failure, had been proven to be effective on PAS, but accompanied with distinct side effect on hemodynamics [14]. From our analysis and the comparisons, ondansetron exhibited a high safety profile.

Study by Browning et al. [17], which found ondansetron could not reduce the incidence of shivering, contributed to the heterogeneity as identified by forest plots and sensitive analysis. The subgroup analysis also confirmed the source of the heterogeneity was from the study (data not show). Many factors could be related to the heterogeneity, the most important one is the definition of PAS the author used [36]. Otherwise, the subjects involved in this study were all female, pregnant, and young, and the anesthetic technique was combined spinal epidural anesthesia, which differed from the others. When omitting this study, homogeneity appeared and the pooled estimate became more significant with a narrower confidence interval.

To our knowledge, this is the first meta-analysis to explore the prophylactic effect of ondansetron on PAS. Additionally, the original studies included in our meta-analysis are all randomized, double-blinded and placebo-controlled trials except one [3] without the mention of blind, and all take PAS as primary outcome. Besides, the negative association between ondansetron and the PAS remained robust and significant in both subgroup and sensitive analyses. Otherwise, we also compared ondansetron with meperidine on the effect of PAS. Moreover, the side effects and dose-dependent effect was also explored.

There are some potential limitations should be considered. Firstly, the test power, to some extent, is limited as confined by the sample size. The individual study in this meta-analysis has a relatively small sample with only one study [17] more than 100 subjects. Secondly, the definitions of PAS vary from each other. Three studies [3,13,17] used a graded scale to evaluate the severity of shivering, and others [14-16] defined PAS as some muscles shivering for minimum 10 or 15 seconds. Thirdly, many included studies only presented data about PAS but not simultaneous side effects (nausea and vomiting, bradycardia, hypertension etc.). And only three studies compared ondansetron with other antishivering drugs, (meperidine, ketamine), we, therefore, could not give a comprehensive evaluation of ondansetron. Finally, dosages of ondansetron were not standard; either 4 mg or 8 mg was administrated in the individual study.

Some directions for future research should be drawn out from current meta-analysis. The adequate sample, comprehensive side effects should be considered in future research, and the effects of various dosage of ondansetron, anesthetic technique and surgical setting on PAS should also be explored. Furthermore, studies aimed to compare ondansetron and other drugs should be conducted to evaluate the clinical and economic efficacy of ondansetron. Finally, the definition of PAS should be standardized.

## Conclusions

Current meta-analysis demonstrated that ondansetron has a preventive effect on PAS without a paralleled side effect of bradycardia.

## Competing interests

The authors declare that they have no competing interests.

## Authors’ contributions

HTT and GZS conceived the study, participated in the design, collected the data, performed statistical analyses, and drafted the manuscript. HTT, GZS and HWY participated in the design, collected the data, and helped to draft the manuscript. JHM, KH and SRL helped to perform statistical analyses and to revise it critically for important intellectual content. All authors read and approved the final manuscript.

## Pre-publication history

The pre-publication history for this paper can be accessed here:

http://www.biomedcentral.com/1471-2253/14/12/prepub
